# Polymorphisms in *Plasmodium vivax* Circumsporozoite Protein (CSP) Influence Parasite Burden and Cytokine Balance in a Pre-Amazon Endemic Area from Brazil

**DOI:** 10.1371/journal.pntd.0004479

**Published:** 2016-03-04

**Authors:** Bruno de Paulo Ribeiro, Gustavo Capatti Cassiano, Rodrigo Medeiros de Souza, Dalila Nunes Cysne, Marcos Augusto Grigolin Grisotto, Ana Paula Silva de Azevedo dos Santos, Cláudio Romero Farias Marinho, Ricardo Luiz Dantas Machado, Flávia Raquel Fernandes Nascimento

**Affiliations:** 1 Programa de Pós- graduação em Ciências da Saúde, Universidade Federal do Maranhão (UFMA), São Luís, Maranhão, Brazil; 2 Laboratório de Imunofisiologia, Universidade Federal do Maranhão (UFMA), São Luís, Maranhão, Brazil; 3 Centro de Investigação de Microrganismos, Faculdade de Medicina de São José do Rio Preto (FAMERP), São José do Rio Preto, São Paulo, Brazil; 4 Departamento de Parasitologia, Universidade de São Paulo (ICB/USP), São Paulo, São Paulo, Brazil; 5 Centro Multidisciplinar, Campus Floresta (Universidade Federal do Acre), Cruzeiro do Sul, Acre, Brazil; 6 Laboratório de Imunologia das Parasitoses (Universidade CEUMA), São Luís, Maranhão, Brazil; 7 Laboratório de Pesquisa Básica em Malária (Instituto Evandro Chagas / Secretaria de Vigilância em Saúde / Ministério da Saúde—IEC/SVS/MS), Belém, Pará, Brazil; Aaron Diamond AIDS Research Center with the Rockefeller University, UNITED STATES

## Abstract

Mechanisms involved in severe *P*. *vivax* malaria remain unclear. Parasite polymorphisms, parasite load and host cytokine profile may influence the course of infection. In this study, we investigated the influence of circumsporozoite protein (CSP) polymorphisms on parasite load and cytokine profile in patients with vivax malaria. A cross-sectional study was carried out in three cities: São Luís, Cedral and Buriticupu, Maranhão state, Brazil, areas of high prevalence of *P*. *vivax*. Interleukin (IL)-2, IL-4, IL-10, IL-6, IL-17, tumor necrosis factor alpha (TNF-α, interferon gamma (IFN-γ and transforming growth factor beta (TGF-β were quantified in blood plasma of patients and in supernatants from peripheral blood mononuclear cell (PBMC) cultures. Furthermore, the levels of cytokines and parasite load were correlated with VK210, VK247 and *P*. *vivax*-like CSP variants. Patients infected with *P*. *vivax* showed increased IL-10 and IL-6 levels, which correlated with the parasite load, however, in multiple comparisons, only IL-10 kept this association. A regulatory cytokine profile prevailed in plasma, while an inflammatory profile prevailed in PBMC culture supernatants and these patterns were related to CSP polymorphisms. VK247 infected patients showed higher parasitaemia and IL-6 concentrations, which were not associated to IL-10 anti-inflammatory effect. By contrast, in VK210 patients, these two cytokines showed a strong positive correlation and the parasite load was lower. Patients with the VK210 variant showed a regulatory cytokine profile in plasma, while those infected with the VK247 variant have a predominantly inflammatory cytokine profile and higher parasite loads, which altogether may result in more complications in infection. In conclusion, we propose that CSP polymorphisms is associated to the increase of non-regulated inflammatory immune responses, which in turn may be associated with the outcome of infection.

## Author Summary

Recent evidences have associated *P*. *vivax* infections with clinical complications, previously only attributed to *P*. *falciparum* malaria. The interaction between host and parasite may contribute to severity of the disease, however, the specific contribution of each factor remains unclear. Previous studies have shown that polymorphisms in *Plasmodium vivax* CSP may interfere in systemic reactions, response to drug treatments, leading to different symptoms as well as humoral responses. In this study, we investigate whether these polymorphisms could influence the parasite load and cytokine profile, which altogether may influence the malaria outcome. In this sense, studies have shown that subjects with high parasitic loads, responding with production of pro-inflammatory cytokines develop a more severe disease. The present data indicate that VK247 variant are associated with significant higher parasite loads and pro-inflammatory cytokine profile compared to VK210 variant. In this regard, this study demonstrates that *P*. *vivax* CSP polymorphisms have systemic effects in the host immune response, and the investigation of immunogenicity of parasite proteins may provide evidences for a better understanding of this infection.

## Introduction

Malaria caused by *Plasmodium vivax* is responsible for approximately 50% of malaria cases that occur outside Africa, predominantly in countries which are in the disease elimination or pre-elimination phase [1]. Once considered clinically mild when compared with *P*. *falciparum* infection, *P*. *vivax* malaria cause debilitating effects that affect social and economic indices of the endemic regions and has been associated with the occurrence of severe cases around the world, including Brazil [2–5]. The mechanisms involved in this process are poorly understood [1,6], however evidences of parasite virulence, host inflammation and parasite burden might play a key role in malaria outcome[7].

Parasite virulence can be determined by genetic variations in *Plasmodium*, particularly on genes encoding immunogenic parasite antigens, such as the repeated portion of the central region of circumsporozoite protein (CSP)[8], which is highly immunogenic, being one of the most studied epitopes of the *Plasmodium* genus [9]. In *P*. *vivax*, CSP exhibits two highly conserved terminal regions (N- and C-terminal) and one variable central region composed by two nonapeptides that repeat in tandem, GDRA(A/D)GQPA and ANGA(G/D)(N/D)QPG, characteristic of the VK210 and VK247 molecular variants, respectively [10,11]. In addition to these two variants, there is also a third variation, *P*. *vivax-*like, whose CSP present the repeated APGANQ(E/G)GGAA sequence [12].

In Brazil, serological and molecular studies have shown the prevalence of these genotypes across the country, especially in the Brazilian Amazon region as confirmed by molecular diagnosis, in a study carried out by Machado and Póvoa [13] in the states of Rondônia, Amapá and Pará. Interestingly, in this work the occurrence of VK210 variant was observed in single infections, whereas the VK247 and *P*. *vivax*-like variants were demonstrated only in mixed infections. The distribution of these variants was reassessed later in five endemic areas of Brazil, and VK210 still the most prevalent variant. However, these results showed a change in the dynamics of the distribution of the VK247 and *P*. *vivax*-like variants because both of them were also observed in single infections [14].

Immunologically, the course of infection by *Plasmodium* depends on the balance in the production of pro- and anti-inflammatory cytokines. In cases where an inflammatory pattern is prevalent, the disease tends to be more severe. In severe malaria, there is an increased level of inflammatory cytokines, such as interferon gamma (IFN-γ, tumor necrosis factor alpha (TNF- α and interleukin (IL)-6 [15,16]. In the other hand, high levels of IL-10 have also been reported in individuals with severe disease and has been associated with cerebral malaria. Nonetheless, the upregulation of IL-10 appears to occur after the increase in inflammatory cytokines, due to a regulatory mechanism, to prevent the exacerbated of inflammatory response and its deleterious effects [15–17].

There are still many questions concerning which factors inherent to the host and the parasite are responsible for increased systemic inflammation, parasite load and worsening of the disease. CSP variants infection may affect drug response, symptom severity and humoral response patterns in the host [13,18,19], however the influence of these variants in the cytokine response and parasite load remains unclear. Thus, the present study aimed to characterize the molecular variants of CSP and to correlate them with the cytokine profile and the parasite load in studied region. This research can demonstrate, even indirectly, differences in the degree of virulence of these *P*. *vivax* variants and clarify the immunological aspects of infection by *P*. *vivax* variants to understand this new scenario associated with the disease.

## Materials and Methods

### Study design, region and population

A cross-sectional study was conducted from January 2011 to February 2013 with *P*. *vivax-*infected patients from São Luís, Cedral and Buriticupu, a pre-Amazon region of the state of Maranhão, which presents a high prevalence of *P*. *vivax* infections. Eligible *P*. *vivax*-infected patients were selected among people who sought medical care at the Center for Infectious and Parasitic Diseases (CREDIP) in São Luís, Unit of Basic Health (UBS) in Cedral and Center of Health UFMA in Buriticupu. Epidemiological questionnaires were applied to every patient who freely agreed to participate the study. The patients group included individuals of both genders who were positive for *Plasmodium vivax* in thick blood smear and negative for *Plasmodium falciparum*, that had not yet received treatment. A control group included healthy individuals who lived in the same area, that were not infected with either *Plasmodium vivax or Plasmodium falciparum*. The thick blood smear malaria diagnosis was further confirmed by nested PCR with species-specific primers based on the *Plasmodium* small subunit ribosomal RNA (ssrRNA) genes, as described by Singh and colleagues[20] with modifications (see S1 Method). The treatment recommended by Brazilian Ministry of Health was guaranteed for all patients, including those who did not agree to participate in the study.

### Collection of plasma, peripheral blood mononuclear cells (PBMC) and erythrocytes

A total of 8 to 10 mL of blood was collected by standardized venipuncture in ethylenediamine tetraacetic acid (EDTA) tubes Vacutainer (Becton Dickinson, San Jose, CA, USA). Then, aliquots obtained from plasma were frozen at -80°C for subsequent evaluation of the cytokines concentration. Approximately 7 mL of the remaining blood was used to the separation process on a Ficoll-Paque PLUS gradient (GE Healthcare, New Jersey, USA) to obtain PBMC. The cells were then resuspended in 1 mL of RPMI 1640 medium (Sigma, St. Louis, USA), supplemented with 2 mM L-glutamine (Sigma, St. Louis, USA), 1% streptomycin (100 μg/mL, Merck), penicillin G (100 U/mL, Sigma, St. Louis, USA) and 10% fetal bovine serum (Sigma, St. Louis, USA). Sample were stained with Trypan Blue and cell counts and viability were accessed using a hemocytometer (Neubauer) chamber. Subsequently, the cells were cultured as posteriorly described. Erythrocytes were stored in two aliquots at -20°C to characterize the molecular variants of *P*. *vivax* and to quantify the parasite load as described later.

### PBMC cultures

PBMCs (2 x 10^6^ cells/mL/well) were plated in duplicates in 48-well flat bottom plates for 48 h at 37°C in a humidified incubator containing 5% CO_2_. After this period, the culture supernatants were collected and frozen at -80°C for subsequent analysis of the cytokines.

### Determination of cytokines by cytometric bead array (CBA)

The concentrations of the IL-2, IL-4, IL-6, IL-10, IFN-γ, TNF-α, IL-17 cytokines were measured using CBA. Th1/Th2/Th17 (Becton Dickinson Biosciences, San Jose, CA, USA). The dosage of TGF-β was carried out using the Single Plex Flex Set (Becton Dickinson Biosciences, San Jose, CA, USA) kit. A total of 50 μL of plasma sample and culture supernatants were analyzed in FACSCalibur flow cytometer (Becton Dickinson, San Jose, CA, USA) previously calibrated with “setup beads” incubated with fluorescein isothiocyanate (FITC) or phycoerythrin (PE) according to the manufacturer`s recommendations. A standard curve was performed for each cytokine. The results were analyzed in the FCAP Array Software (Becton Dickinson, San Jose, CA, USA) and the values were expressed in pg/mL for each cytokine.

### Genotyping of the CSP of *P*. *vivax*

Plasmodial DNA was extracted from an aliquot of erythrocytes using the *Easy*-*DNA* Kit (Invitrogen, Carlsbad, CA, USA), according to the recommendations of the manufacturer. The amplification was performed according to the description by Cassiano and colleagues, with modifications [21]. A reaction mix with a final volume of 25 μL was obtained: *P*. *vivax* DNA, 1 X PCR buffer (20 mM Tris–HCl pH 8.4, 50 mM KCl), 1.6 mM MgCl, 0.2 mM of each dNTP, 0.2 μM of each primer (5’ AGGCAGAGGACTTGGTGAGA 3’ and 5’CCACAGGTTACACTGCATGG 3’) and 1 U of Taq Platinum (Invitrogen, Carlsbad, CA, USA).

The reaction was performed in a thermocycler (DNA MasterCycler, Eppendorf, Germany) as follows: an initial cycle of 94°C for 15 min, followed by 30 cycles of 94°C for 1 min, 58°C for 1 min and 72°C for 1 min, with a final extension at 72°C for 10 min. As a positive control, three plasmids were used, containing a gene insert of the repeat portion of CSP amplified from the VK210, VK247 and *P*. *vivax*-like variants (BlueScript, Stratagene, La Jolla, USA). For the negative control of the reaction, sterile water was used.

The *P*. *vivax* CSP variants were characterized by the analysis of the PCR-restriction fragment length polymorphism (PCR-RFLP), following the method described by Cassiano and colleagues [21]. The digestion reaction was performed in a final volume of 20 μL: 10 U of AluI (Invitrogen), 2 μL of the enzyme reaction buffer, 10 μL of the PCR product and 7 μL of sterile DNAse-free water. The reactions were performed in a water bath at 37°C overnight.

### Quantification of the parasite load by real-time PCR (qPCR)

Plasmodial DNA was extracted from an aliquot of the blood using the QIAamp DNA Mini Kit (QIAGEN, Hilden, Germany), according to the recommendations of the manufacturer. The qPCR was developed according to Gonçalves et al. [22], using the commercial kit Maxima SYBR Green (Fermentas, Lithuania) and preparing a reaction mix with a final volume of 15 μL: 7.5 μL of Maxima SYBR Green master mixture, 0.5 μM of each primer, 4.0 μL of DNAse-free water and 2 μL of genomic DNA. The P1 genus-specific primer (5’-ACGATCAGATACCGTCGTAATCTT-3’) was combined with a species-specific oligonucleotide primer for *P*. *vivax*, V1 (5’-CAATCTAAGAATAAACTCCGAAGAGAAA-3’). These primers amplified a 100-bp species-specific fragment of the 18S rRNA gene.

The assays were performed in triplicate in a Mastercycler Realplex Gradient Thermal Cycler (Eppendorf, Hamburg, Germany) and consisted in the denaturation of the template DNA at 95°C for 10 minutes and 40 cycles of 15 seconds at 95°C and 1 minute at 60°C, acquiring fluorescence at the end of each step of the extension. The amplification was immediately followed by a dissociation curve that consisted of 15 seconds at 95°C, 15 seconds at 60°C and one gradual temperature increase of 0.2°C.s^-1^ up to 95°C, acquiring fluorescence at each temperature transition. The result obtained by the software is expressed as the number of copies of the plasmodial genome in a DNA sample, which allows the estimation of the corresponding number of parasites in the sample. The sequence target possessed a length of about 100 base pairs. For quantitation of parasitemia it was used as standard an amplified fragment with oligonucleotide species-specific, purified and cloned into plasmid vector (pGEM T-Easy, Promega), used to transform bacteria DH10B strain. After confirmation of the sequence of cloned recombinant plasmids by sequencing, the solutions with the correct sequence were measured by spectrophotometry and the number of copies per μL of the target sequence was calculated. It was constructed a curve with 10 points from the serial dilution (dilution factor: 10) of the plasmidial solution. The first point contained 1.468x10^9^ copies of the target sequence and the qPCR quantification limit was 0.2 parasites/μL of blood.

### Statistical analysis

The statistical analyses were performed using GraphPad Prism 5.0 and Stata 12 softwares. The gender proportion on the variants was compared using Fisher’s exact test. After the normality test of D’Agostino and Pearson, the differences observed in the cytokine profile were evaluated using the two-tailed Mann-Whitney test. For the correlation analyses, the values were log transformed, and the Spearman test was used to evaluate individual cytokines and parasite load associations. To verify the multiple association of cytokines with parasite load the multivariable linear regression model was used. Cytokines and parasite load data were expressed by median (interquartile range-IQR). Differences were considered significant when *p* values ≤ 0.05.

### Ethics considerations

The present study was approved by the Ethics and Research Committee of the Universidade Federal do Maranhão (Protocol no. 23115 008013/ 2010–07). All the study participants signed a written informed consent or had their legal guardians, if they were underage.

## Results

### Study samples and population

A total of 33 individuals tested positive for *P*. *vivax*, however 8 were excluded because they had already started treatment. The 25 remaining patients were from the pre-Amazon region of Maranhão, of both genders (24% were women and 76% were men) with mean of 32.9 years old (range 3 and 58 years). All patients were identified infected only with *P*. *vivax*. The healthy group consisted of nine individuals from pre-Amazon region, state of Maranhão, Brazil, which had similar profile to patients group, with mean of 29.4 years old (23% women and 77% men).

### Genotyping of the CSP

The most prevalent genotype was VK210 (56%), followed by mixed infection (VK210/VK247) (24%) and VK247 (20%). None of the patients had the *P*. *vivax*-like variant. VK210, VK247 and mixed infection groups had a similar mean of age (38.7, 44.4 and 43.4 years, respectively). The gender difference was not statistically significant among VK210 and VK247 groups (85.8% and 80% of males, respectively) (*p* = 1.0). On the other hand, mixed infection group had a half of male patients (50%), but also was not statistically different of VK247 (*p* = 0.54) and VK210 (*p* = 0.13) proportion.

### Plasma cytokine profile

**Fig 1:**
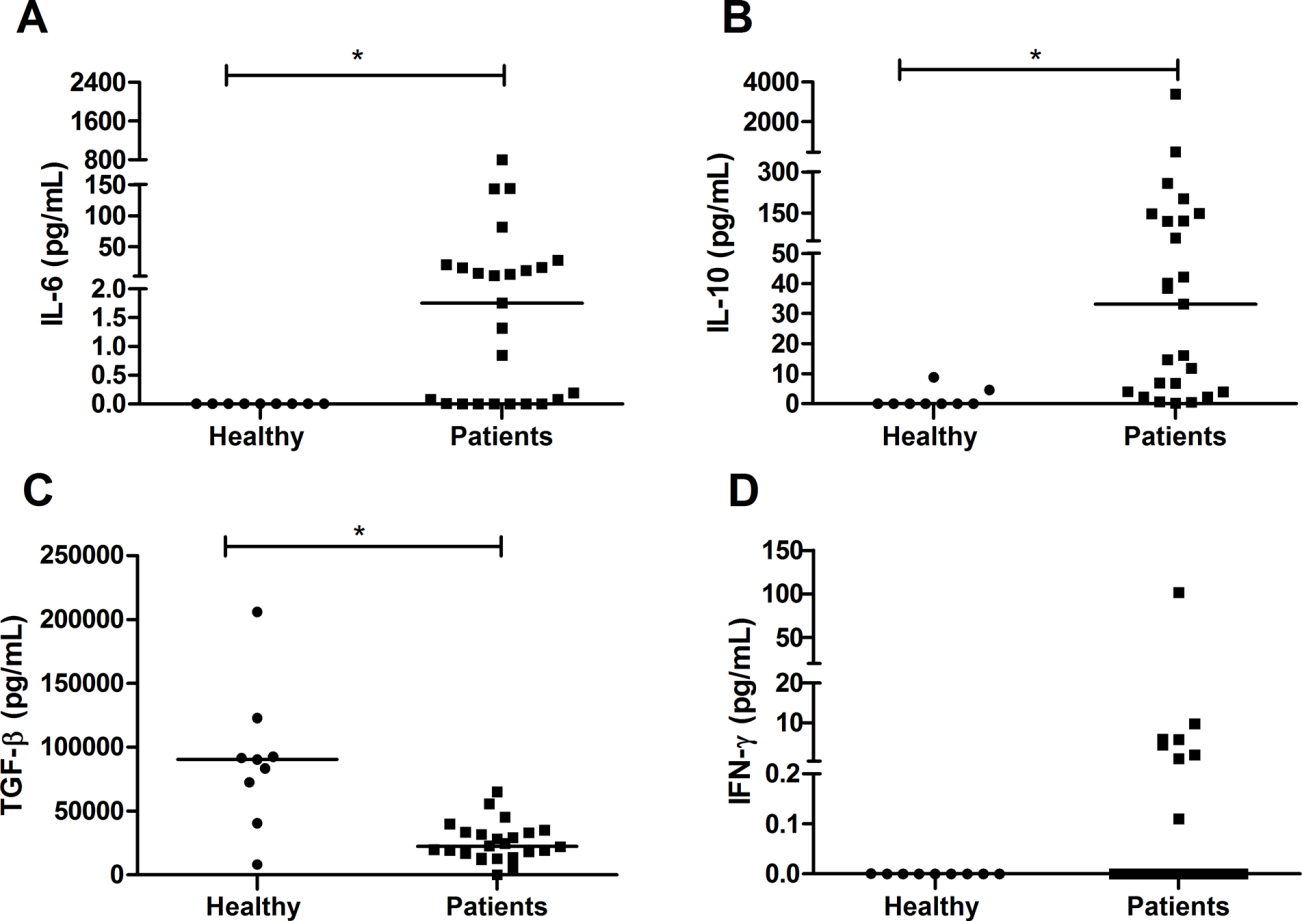
Cytokine profile in the plasma of patients infected with *P*. *vivax*. Cytokines were measured by CBA. A: Interleukin 6; B: Interleukin 10; C: Transforming growth factor beta; D: Interferon gamma. The bar represents the median of the group, n = 25 infected patients and 9 healthy patients (A,B,D) and n = 24 infected patients and 9 healthy patients (C). *P* values were determined by nonparametric Mann-Whitney U tests (*p<0.001).

The plasma levels of IL-2, IL-4, TNF-α and IL-17 were very low or undetectable in patients and healthy group. The concentrations of IL-6 (median of 1.75 pg/mL; IQR 0.005–18.7 pg/mL) (Fig 1A) and IL-10 (median of 33.05 pg/mL; IQR 3.9–135.4 pg/mL) (Fig 1B), in turn, were higher in individuals infected by *P*. *vivax* (*p* = 0.0009 and *p* = 0.0002, respectively) when compared to healthy group (median of zero for both IL-6 and IL-10). Conversely, the TGF-β concentration was lower in infected individuals (median of 22417 pg/mL; IQR 14341–33508 pg/mL) (*p* = 0.0009) when compared to healthy group (median of 90507 pg/mL; IQR 56580–107531 pg/mL) (Fig 1C). Only eight patients had detectable IFN-γ concentrations with a median of approximately zero; however, there was no significant difference compared to the healthy group (Fig 1D).

### Relation between cytokines and parasite load

**Fig 2:**
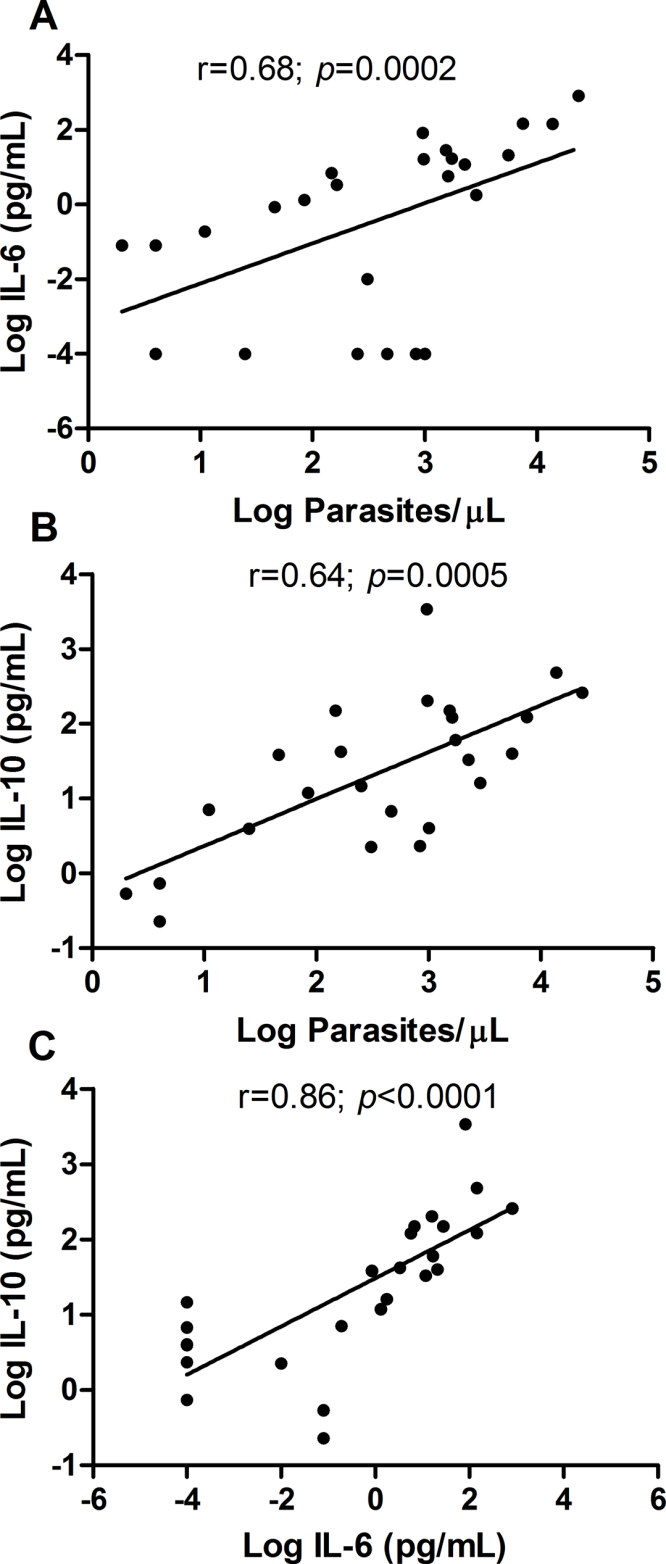
Correlation between parasite density and the cytokine profile. The *x* axis indicates Log Parasites/μL (A and B) or Log IL-6 (pg/mL) (C); the *y* axis indicates Log Interleukin 6 (pg/mL)(A), Log Interleukin 10 (pg/mL) (B) and (C). Each point represents the relative parasitaemia and cytokine responses of a single patient (A and B) or represents the relative IL-10 and IL-6 (C) responses of a single patient, n = 25 infected patients. The correlations were determined by Spearman’s correlation tests.

The median parasite load quantified by qPCR was 837 parasites/μL (IQR 65–2000 parasites/μL), with positive correlation both with IL-6 (r = 0.68; *p* = 0.0002) and IL-10 (r = 0.64; *p* = 0.0005) (Fig 2A and 2B), indicating that these two cytokines tend to increase in response to a raise in parasitaemia. In addition to their correlation with the parasite load, IL-6 and IL-10 also had a positive correlation with each other (r = 0.86 and *p*<0.0001) (Fig 2C). The correlation between the remaining cytokines was not performed due to very low or undetectable concentrations in the plasma of patients.

However, the multiple linear regression analyses among the IL-10, IL-6 and parasite load showed that only IL-10 keeps this association with parasite load (r = 0.79 and *p* = 0.005) (Table 1).

**Table 1:** Multivariable analysis of correlation among cytokines and parasite load.

Factors	Log parasite load		
	Coeficient	*p* value	95% Conf. interval
**Log IL-6**	-0.002	0.980	-0.230; 0.224
**Log IL-10**	0.795	0.005	0.267; 1.324

### Influence of the CSP genotypes in the cytokine profile and in the parasite load

**Fig 3:**
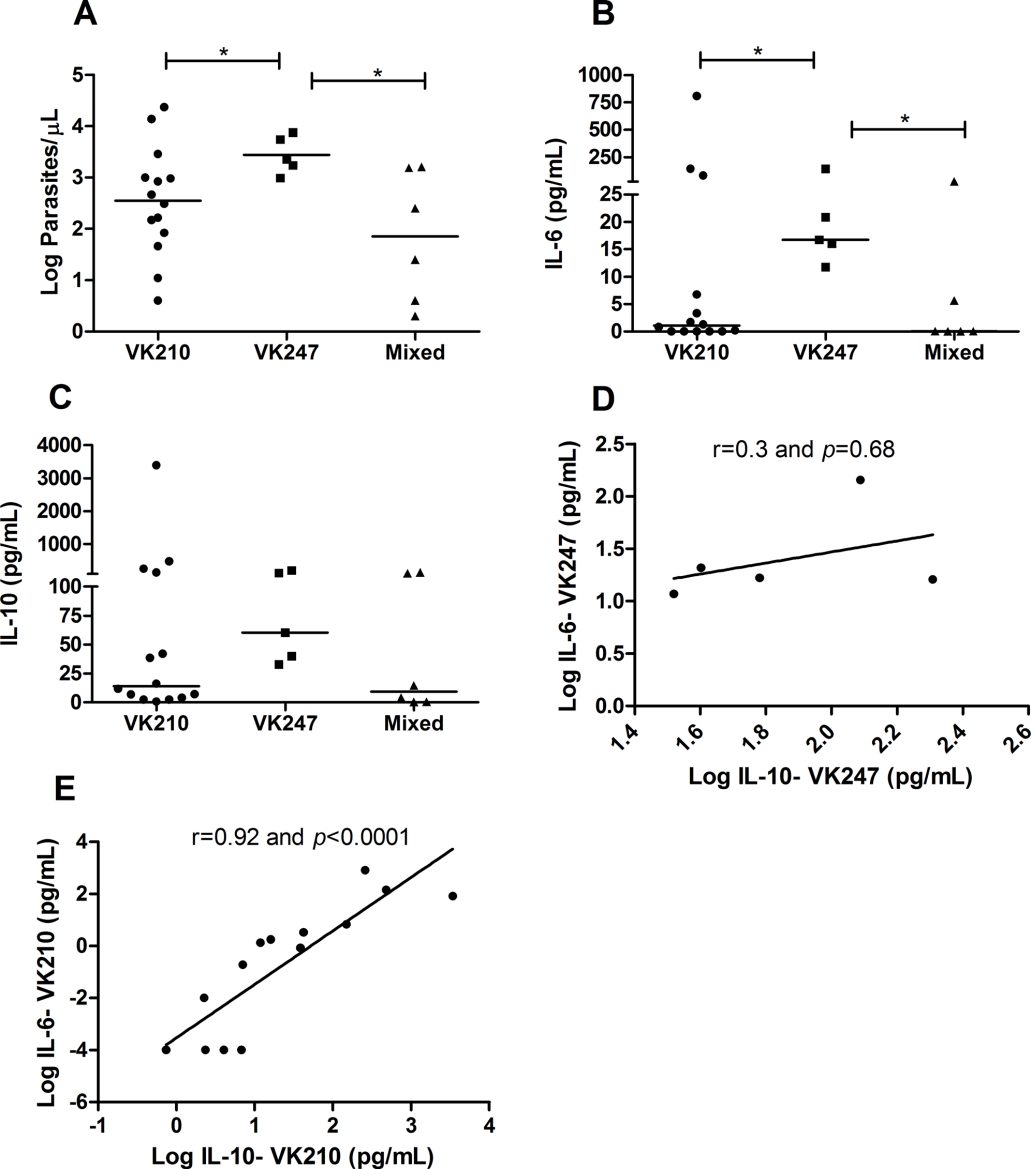
Cytokine profile and parasite density in *P*. *vivax* circumsporozoite protein (CSP) genotypes. Cytokines were measured by CBA and parasite density was determined by real-time PCR. A: Parasite density (Log parasites/μL); B: Interleukin 6 (pg/mL); C: Interleukin 10 (pg/mL); The bar represents the median of the group, n = 25 patients. *P* values were determined by nonparametric Mann-Whitney U tests (*p≤0.05). In correlations analyses, the *x* axis indicates Log IL-10 patient concentrations (pg/mL) in VK247 (D) or VK210 (E); the *y* axis indicates Log IL-6 patient concentrations (pg/mL) in VK247 (D) or VK210 (E). Each point represents the relative IL-10 and IL-6 responses of a single patient. Correlations were determined by Spearman’s correlation tests.

The parasite loads were higher in individuals infected by VK247 (median of 3.35 log parasites/μL; IQR 3.1–3.8 log parasites/μL) compared to patients infected by VK210 (median of 2.5 log parasites/μL; IQR 1.8–3.1 log parasites/μL) (*p* = 0.05) and patients with mixed infection (median of 1.8 log parasites/μL; IQR 0.52–3.1 log parasites/μL) (*p* = 0.01) (Fig 3A). The same fact was observed regarding IL-6 concentrations, which levels were higher in patients with VK247 (median of 16.7 pg/mL; IQR 13.9–82.39 pg/mL) compared to patients with VK210 (median of 1.08 pg/mL; IQR 0.0001–25.54 pg/mL) (*p* = 0.04) and to those with mixed infections (median of 0.08 pg/mL; IQR 0.0001–11.25 pg/mL) (*p* = 0.05) (Fig 3B).

The variants did not influence the IL-10 concentrations (Fig 3C), but in patients infected with VK210, the IL-10 levels correlated with IL-6 concentrations (r = 0.92 and *p*<0.0001) (Fig 3E). However, in patients with VK247, this was not observed and no correlation between the two cytokines was found (r = 0.3 and *p* = 0.68) (Fig 3D), which indicates that the increase in inflammatory cytokines was not followed by the regulatory effect of IL-10 in these patients.

### Cytokine profile in the culture supernatant

**Fig 4:**
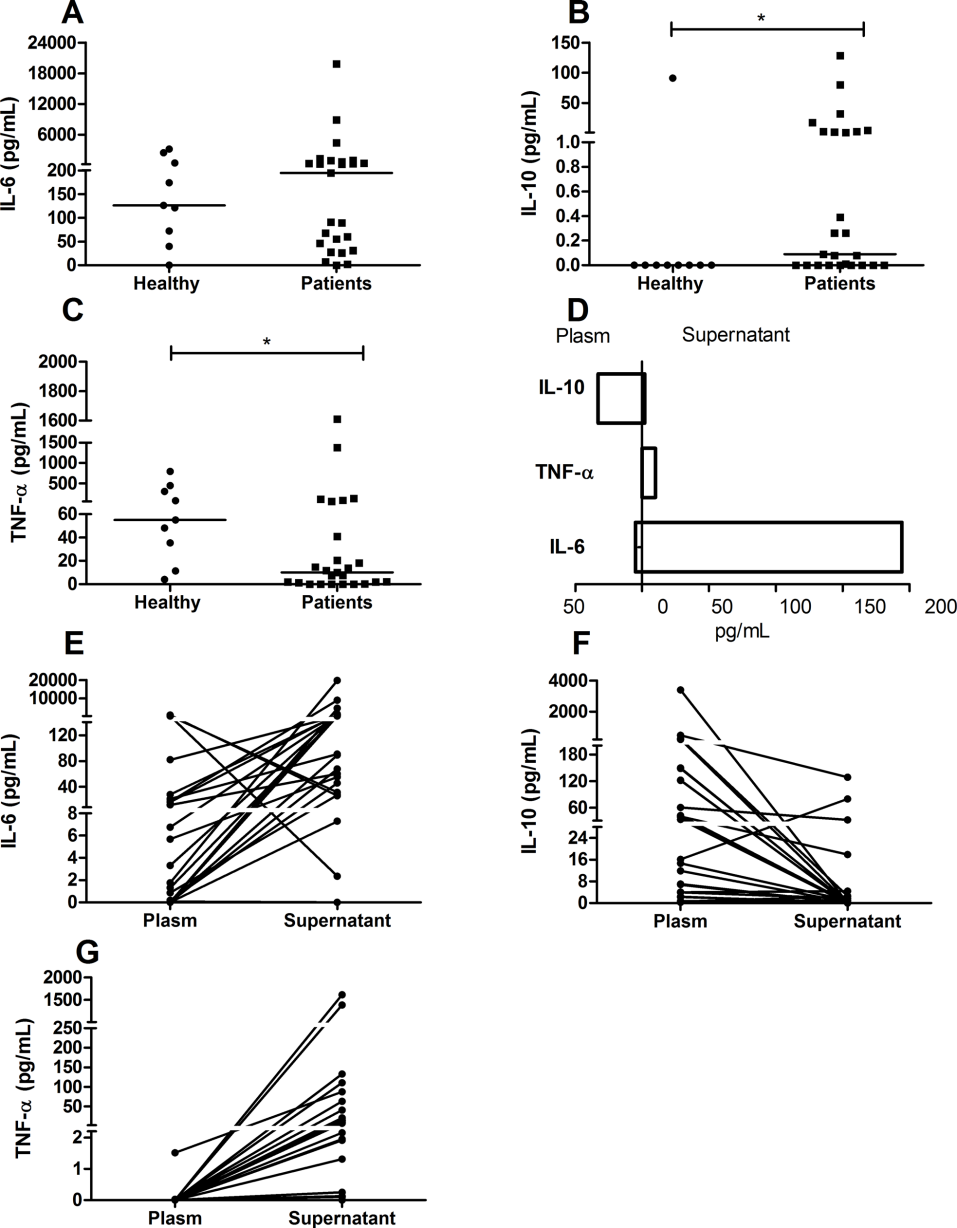
Cytokine profile in supernatant of patients infected with *P*. *vivax*. Cytokines were measured by CBA. A: Interleukin 6 (pg/mL); B: Interleukin 10 (pg/mL); C: Tumor necrosis factor alpha (pg/mL). The bar represents the median of the group, n = 25 patients and 9 healthy group. *P* values were determined by nonparametric Mann-Whitney U tests (*p≤0.05). In E, F, and G, each point represents the relative plasma/supernatant IL-6 (E), IL-10 (F), and TNF-α (G) responses of a single patient. In D, the bars represent the median of each cytokine in the plasma or supernatant.

In the PBMC culture supernatants, only the IL-10 (median of 0.09 pg/mL; IQR 0.0001–2.5 pg/mL) and TNF-α (median of 10.02 pg/mL; IQR 0.78–52.06 pg/mL) concentrations were significantly different compared to the healthy group (median of zero for IL-10 and 54.98 pg/mL with IQR 23.3–375.6 pg/mL for TNF-α; IL-10 was higher (*p* = 0.03) (Fig 4B) and TNF-α was lower (*p* = 0.03) (Fig 4C). In contrast to the results observed in the plasma, IL-6 produced by PBMCs of patients (median of 194 pg/mL; IQR 38.3–808.3 pg/mL) did not show a significant difference from the healthy group (median of 126 pg/mL; IQR 55.8–1472 pg/mL) (Fig 4A).

Interestingly, a different cytokine profile was observed between plasma and the PBMC cultures supernatants. The median IL-10 concentration was 367 times higher in the plasma from patients when compared to that of the culture supernatant. The concentration of the inflammatory cytokines TNF-α and IL-6 were higher in the culture supernatant than in the plasma, and IL-6 reached 111 times higher concentrations in the supernatant (Fig 4D). Most patients had an increased IL-6 concentration in the culture supernatant compared to that in the plasma (Fig 4E). Individuals who exhibited a greater IL-10 response in the plasma (above 30 pg/mL) had cells that produced lower amounts of the same cytokine in culture (below 8 pg/mL) (Fig 4F). In addition, patients with low levels of IL-10 in the plasma maintained this low baseline production in culture supernatant. Altogether, these facts explain the small median value found for this cytokine in the culture supernatant. Little or no TNF-α production was detected in the plasma of infected patients, however an increase in production was observed the culture supernatants (Fig 4G).

## Discussion

*P*. *vivax* sporozoites are covered with CSP, a highly immunogenic protein, which is involved in invasion mechanisms into hepatocytes. In the present study, it was shown that the CSP polymorphisms are determinants for both the cytokine balance and the parasite load in vivax malaria. Further, the VK247 variant induced a more prominent inflammatory profile and the highest parasite loads, suggesting that these CSP polymorphisms have systemic effects changing variables that may influence the course of infection.

Initially, it was observed that patients infected with *P*. *vivax* showed increased plasma IL-10 and IL-6 levels which was associated with the parasite load. This result corroborates with other authors who demonstrated that increased concentrations of IL-6 and IL-10 producing cells, were associated with increased parasitaemia [16,23]. Our results support the notion that in the initial moments of the infection, the IL-6 concentration increased as a response to increase in parasite load and is subsequently controlled by IL-10 secretion as has been discussed for other authors [15–17]. This hypothesis was reinforced in this study by the multivariable regression analyses, which showed that, despite the individual correlation of parasite load with IL-6, only IL-10 is associated to parasite load in this context. Thus, we suggest that the increase of IL-6 induces a counter-regulatory IL-10 production, which, in turns, is crucial to the balance of the parasite-host interaction.

It is important to emphasize that the severity of the malaria is intrinsically related to inflammatory reaction and, despite the fact that IL-10 prevents the deleterious effects caused by a exacerbated inflammatory response [23,24], it may contributes to the maintenance of the parasite in the host, in an equilibrated relation. Hence, it is reasonable to think that the malaria outcome depends on the ability of parasite to regulate the inflammatory response by induction of IL-10 production. This hypothesis is reinforced by a murine model where it was demonstrated that IL-10 depleted mice are able to control parasite replication during *P*. *chabaudi* AS infection, however, they develop a severe malaria mediated by inflammatory cytokine action [25,26].

The cytokines IL-10, IL-6 and TNF-α were detected in the PBMC culture supernatants, however an inversion in the cytokines pattern was observed in relation to plasma levels (Fig 4D). The IL-10 concentration was higher in plasma than in the supernatant (Fig 4E), in the other hand inflammatory cytokines such IL-6 and TNF-α were higher in the supernatant (Fig 4E and 4F). These results suggest that despite the PBMC from patients produced more IL-10 than healthy individuals (Fig 4B), the IL-10 production was almost abolished in the absence of systemic influence of parasite (Fig 4D). Based on that we suppose that the regulatory pattern observed *in vivo* is not necessary *ex vivo* anymore, despite the presence of high levels of IL-6, differently from that observed *in vivo*, where the regulatory response was important to control IL-6.

Given this regulatory response that seems to occur *in vivo*, we investigated the influence of CSP gene polymorphisms on the plasmatic cytokine profile. The present study provided evidences, even that indirectly, of differences in the modulation of host response and malaria outcome among variants of this protein. In this study, there was a prevalence of VK210 in single infections, what corroborate with other studies performed in the same region [14,27]. This demonstrates that there was no dominance alternation among the variants along years in this endemic area.

It has already been shown that individuals with severe vivax malaria have an imbalance in their cytokine response toward an inflammatory profile and also have higher parasitaemia compared to those with uncomplicated malaria [15]. Based on this fact, we investigated the influence of the variants on the parasite load of the individuals. The results of Fig 3A show that patients infected with VK247 exhibited a significantly higher parasite load than those with VK210 and mixed infections. This result differs from that found by Machado and Póvoa [13], who observed the greatest parasitaemia in individuals infected by VK210. This difference may be because the authors not found patients with VK247 in single infections, what impairs to compare the results. A possible hypothesis for VK247 higher parasite loads is the “immune selection” occurrence, since the constant VK210 prevalence over all these years in analyzed region may have generated high anti-VK210 antibody titers, hindering the infection establishment by this variant and facilitating by VK247. Even though we did not conduct the antibodies research, it is known that in Brazilian Amazon endemic areas there is a smaller antibody response to VK247 in the presence of VK210 [18].

Even though CSP expression occurs only in liver stage [28] the immunological response to this protein reflects in blood stage of the parasite, since it is a target not only to humoral response but also to a protective cellular response [29]. Moreover, in a murine model of natural infection, it was demonstrate that CSP-specific CD8+ T cells were primed by dendritic cells not only locally in liver but also in draining lymph nodes [30]. Beside this, it was found in a murine model that sporozoite antigen persists for over 8 weeks after immunization and remains being presented to naive cells, including those that are recently recruited from thymus [31].

In order to investigate the systemic influence of CSP variants, we performed correlation analysis of these variants with the plasmatic cytokines. A significant increase of IL-6 in patients with VK247 infection was observed when compared to VK210 and mixed infections (Fig 3B). On the other hand, there was no difference in IL-10 production among the groups (Fig 3C). Additionally, in patients with VK210 infection there was a significant positive correlation between IL-6 and IL-10, which suggests that the increase in inflammatory cytokines was followed by increase in the regulatory response in the same patients (Fig 3E), correlation that was not observed in VK247 infected patients (Fig 3D). These findings demonstrate that VK247 may cause a sustained and stronger inflammatory response than VK210 since this response was not followed by IL-10. Due to this absence of association, we suggest that the necessary equilibrium to control the infection is not present in the VK247, since there is a high inflammatory response and parasite load, without association to the counter-regulatory effect of IL-10 cytokine. Based on this context we suggest that VK247 may induce a more severe infection pattern.

Despite the apparent association of plasmatic inflammatory pattern to the variant, there was no significant difference in IL-6 and IL-10 in PBMC supernatant. Besides, there was no correlation between these cytokines in each variants groups (S1 Fig). This result from plasma and PBMC cultures demonstrates that the CSP variants may be, at least partially, different in their ability to induce initial inflammatory response.

In conclusion, we showed for the first time that *P*. *vivax* CSP polymorphisms may have systemic effects that influence the cytokine profile and parasite load. It is important to emphasize that VK247 is not common in single infection in the pre-Amazon region [13,14,27]. However, in our study, the sample of VK247 in single infection was representative since we had a high percentage (20%). Our findings represent an important step for better understanding the new scenario related to vivax malaria and shows that polymorphisms in immunogenic proteins of the parasite may be crucial to better understanding this infection and predict malaria outcome.

## Supporting Information

S1 Fig Cytokine profile in supernatant according circumsporozoite protein (CSP) genotypes. Cytokines were measured by CBA. A: Interleukin 6 (pg/mL); B: Interleukin 10 (pg/mL); The bar represents the median of the group, n = 25 patients. *P* values were determined by nonparametric Mann-Whitney U tests. In correlations analyses, the *x* axis indicates Log IL-6 supernatant concentrations (pg/mL) in VK210 (C) or VK247 (D); the *y* axis indicates Log IL-10 supernatant concentrations (pg/mL) in VK210 (C) or VK247 (D). Each point represents the relative IL-10 and IL-6 responses of a single patient. Correlations were determined by Spearman’s correlation tests.
(TIF)

S1 Method Nested PCR for *Plasmodium vivax* and *Plasmodium falciparum* characterization. To confirm the thick blood smear diagnosis was performed a nested PCR with species-specific primers based on the *Plasmodium* small subunit ribosomal RNA (ssrRNA) genes.
(DOCX)

S1 Strobe Checklist of items that should be included in cross-sectional studies. Detailed list of each item of a cross-sectional study and its location in the manuscript.
(DOCX)

S1 Dataset Data underlying the findings described in manuscript. Complete and individual data about the cytokine concentrations, parasite load and characterization of CSP variants.
(XLSX)
