# Supplementary material for: Polymorphisms in Plasmodium vivax Circumsporozoite Protein (CSP) Influence Parasite Burden and Cytokine Balance in a Pre-Amazon Endemic Area from Brazil
Source: PLoS Negl Trop Dis. 2016 Mar 4;10(3):e0004479. doi: 10.1371/journal.pntd.0004479 (PMC4778932; doi:10.1371/journal.pntd.0004479)
Supplement: S1 Method — To confirm the thick blood smear diagnosis was performed a nested PCR with species-specific primers based on the Plasmodium small subunit ribosomal RNA (ssrRNA) genes. (DOCX) [file pntd.0004479.s002.docx]

**Supplementary Method**

Nested-PCR

The patients group included individuals who had positive thick blood smear for *Plasmodium vivax* and negative for *Plasmodium falciparum*. To confirm the thick blood smear diagnosis was performed a nested PCR with species-specific primers based on the *Plasmodium* small subunit ribosomal RNA (ssrRNA) genes, as described by Singh and colleagues[19] with modifications, as follow:

Plasmodial DNA was extracted from an aliquot of the concentrate of erythrocytes using the QIAamp DNA Mini Kit (QIAGEN, Hilden*,* Germany), according to the recommendations of the manufacturer. The amplification was performed according to the description by Singh and colleagues., with modifications[1]. A reaction mix with a final volume of 25 µL was obtained: 5 µL of DNA template, 0.25 µM of each primer (rPLU 1 and rPLU 5), 4 mM MgCl_2_ (Fermentas, Lithuania), 10 X PCR buffer (10 mM Tris–HCl, 50 mM KCl, 0.08% v/v) (Fermentas, Lithuania), 0.12 mM of each dNTP (Roche) and 0.16 U of Taq DNA polymerase (Fermentas, Lithuania).

Nest 1 amplification conditions were as follows: step 1, 95ºC for 4 min; step 2, denaturation at 95ºC for 30 sec; step 3, annealing at 55ºC for 1 min; step 4, extension at 72ºC for 90 sec; repeat steps 2–4 35 times, and step 4 for 5 min. One microliter (1 µL) of the nest 1 amplification product served as the DNA template for the nest 2 amplifications. Nest 2 amplification conditions were identical to those of nest 1 except that the annealing temperature in step 3 was 58ºC for the species-specific primers (rFAL 1 and 2, rVIV 1 and 2). The PCR products of nest 2 amplifications were analyzed by gel electrophoresis and staining with bromophenol blue.

The primers used were:

rPLU 1: 5’- TCA AAG ATT AAG CCA TGC AAG TGA-3’

rPLU 5: 5’-CCT GTT GTT GCC TTA AAC TCC-3’

rFAL 1: 5’- TTA AAC TGG TTT GGG AAA ACC AAA TAT ATT-3’

rFAL 2: 5’- ACA CAA TGA ACT CAA TCA TGA CTA CCC GTC-3’

rVIV 1: 5’- CGC TTC TAG CTT AAT CCA CAT AAC TGA TAC-3’

rVIV 2: 5’- ACT TCC AAG CCG AAG CAA AGA AAG TCC TTA-3’

Reference

1.Singh B, Bobogare A, Cox-Singh J, Snounou G, Abdullah MS, Rahman HA. A genus- and species-specific nested polymerase chain reaction malaria detection assay for epidemiologic studies. Am J Trop Med Hyg. 1999;60: 687–692.
